# Annotations

**Published:** 1899-11-18

**Authors:** 


### ANNOTATIONS.

### Country Homes for Working People.

THE admirable address given by Lord Rosebery at the opening of some municipal dwellings for working people at Shoreditch last Monday formulates and gives point to a great London want, to which we have many times drawn attention. London consists of a number of municipalities closely packed together, and thus deprived of all power of expansion. In this the metropolitan differ absolutely from the provincial municipalities; for, while the latter can expand in proportion to their growth, the London parishes can but overflow into one another. This is the key to much of the difficulty which is being felt in almost every parish in regard to the healthy housing of its working-class population. If a parish is to provide for its own increase it can only do so by increasing still more the overcrowding which is at the root of the whole sanitary difficulty in London. At the present no parish, and not even

**ANNOTATIONS**—*continued.*

London as a whole, has power to buy land in the country outside its boundaries, and there erect villages and subsidiary towns of workmen's dwellings. If it is to do anything for its working-class population it must do it within its own borders. Yet, as Lord Rosebery says: "You may buy land outside the County of London for lunatic asylums. . . . You have power to expatriate the ruin and wreck of your population to the great asylums in the middle of the rural districts. Why, on the other hand, should you not have the power . . . to lodge not merely your lunatics and invalids, but also your intelligent artizans with their large families outside the county of London, so that they may be brought up under healthier conditions and have easy access to their work through the railway trains." When one considers the rate at which the population of London grows—something like 60,000 every year—one sees the impossibility of the various parishes, hemmed in as they are by other parishes, or even of the County Council, limited as it is by its boundary lines, providing for this teeming multitude in proper and healthy fashion within their own areas. Lord Rosebery very properly pointed out that even in such a parish as Shoreditch, where so much is being done, "there is, and always will be, one great flaw. The inhabitants of the wretched tenement are turned out and admirable new dwellings are erected, but the inhabitants of these new dwellings are not the people who were dispossessed. They cannot be. As vestries . . . begin operations of this kind . . . they will have indeed an improved workman's city growing up in the heart of London, but they will have equally pushed backward, backward, and backward the residuum of misery and crime which in the long result they will have to deal with." "I believe," said Lord Rosebery, "that if the county of London had the power to purchase tracts of land—for example in Essex, where much of the land has been turned out of cultivation, owing to agricultural distress—you would have marched, not the whole way, because you would not have dealt with the residuum of which I have spoken, but you would have gone a long way to deal with the big problem of London habitation." There can be no doubt that Lord Rosebery has put forward a proper and a statesmanlike suggestion for dealing with a great and a growing difficulty.

### Tinned Surgical Dressings.

There seems a great future for tinned surgical dressings. Where operations are done amid such conditions as to allow of the sterilisation of all dressings by heat such luxuries are not necessary, but when there is any doubt in regard to the surroundings in the midst of which one may have to work, every means by which one can ensure a supply of dressings and artificial sponges the absolute sterility of which can be relied upon is an untold advantage. There seems no better way of preserving such things in all their original purity than that adopted by Mr. Treves in regard to the dressings taken out by him to South Africa, namely, having the quantity of material which is likely to be required for each operation hermetically sealed up in a separate tin box. The artificial sponges, ligatures, &c., together with a bottle of phenol sufficient to make carbolic solution for the instruments, are enclosed in a tin box, for all the world like a very large box of sardines, and sterilised by heat. By tearing off a strip of tin which covers the joint between box and lid the latter can be removed and made to serve as a sterilised tray for instruments. Useful as these tinned surgical dressings are likely to be in South Africa there seems a great opening for their employment in civil life also. It is very difficult for a practitioner to deal on aseptic principles with surgical emergencies which only arise occasionally. The trouble lies in the sponges, ligatures, and dressings; but such things done up in tins and always ready are likely to prove as useful a resource to the general practitioner in case of unexpected operation as the tinned tongues, sardines, and other comestibles are to the country housekeeper on the arrival of unexpected guests.

### A Profession Forging Fetters for Itself.

At the suggestion of its medical officer Norwich is about to try the experiment of the voluntary notification of phthisis, and the Health Committee is going to pay the fee of 1s. for each notification received. There is but little doubt that, given a judicious medical officer and an intelligent sanitary committee, notification of everything from tuberculosis to dust heaps is of advantage to the public. The Health Committee and their medical officer are, then, quite right in endeavouring to organise this experiment; but whether it is wise for the medical profession to fall in with the suggestion in its present form is quite another affair. It must be distinctly understood that this is not a matter of philanthropy. If the sanitary authority wants the information it ought to pay for it according to the standard fees fixed by the Notification Act, and not with a miserable shilling. But even if the fees offered were better, we doubt very greatly whether medical men will do wisely in falling in with such an arrangement. The saddle will always be put upon the willing horse, and when put on it will not readily be taken off again. A shilling is a ridiculous remuneration to offer for the extremely responsible work of certifying whether or not a patient is suffering from phthisis, and if it is said that it is done for the public good, then we answer that, if so, the public ought to pay for it. By giving such cheap service the profession is forging fetters for itself. We may be quite sure that, if these things are done voluntarily now, they will be imposed upon us by law later on; and that, if we voluntarily lower the fee which at present is payable for notification, we shall before long be made to notify without any recompense at all. If in return for the long course of study which we are compelled to undertake, and the strict examinations which we are compelled to undergo before we are recognised by law as qualified medical practitioners, we received some sort of protection from the competition of unqualified men—if, that is, the Government would prohibit and put a stop to practice by unqualified persons in the same sense that it prohibits and prevents the sale of spirits by unlicensed people, then there would perhaps be some reason in demanding that we should undertake some public service in return for the protection so afforded. But as things are we owe the public nothing, and if the public wants any service at our hands it ought to pay, and to pay well.

